# Keeping AI in medicine and radiology within the framework of scientific method: measuring to close the epistemic gap

**DOI:** 10.1186/s13244-025-02171-7

**Published:** 2025-12-22

**Authors:** Filippo Pesapane, Francesco Sardanelli

**Affiliations:** 1 Breast Imaging Division, Radiology Department, IEO European Institute of Oncology IRCCS, Milan, Italy; 2 Lega Italiana per la Lotta contro i Tumori (LILT) Milano Monza Brianza, Milano, Italy

The classical scientific method—systematic observation, question formulation, hypothesis construction, experimental testing, analysis (including rejection or refinement), conclusion, and transparent communication—structures reliable inference in medicine, based on the long way of development of modern science. Contemporary artificial intelligence (AI) and, in particular, foundation/large language models, seem to be inverting this logical one-way order by proposing “end-to-end” systems and supplying post hoc rationales. We argue that this “black-box acceleration” risks erasing intermediate epistemic steps unless development and evaluation are explicitly re-embedded in the workflow. “Explainable” AI does not guarantee recovery of reasoning because of an epistemic gap: explanations are constrained to human clinical concepts even when a model’s decision basis may be non-human yet causally valid, non-human and spurious, or human-readable but only post hoc. Of note, medical imaging AI studies show saliency instability, shortcut learning, and distributional fragility, especially when a system is translated to new/unknown contexts. Training and validating systems on the deployment population within a defined technical context reduces domain mismatch and improves calibration but can entrench local biases and impair portability to other populations or different technology systems. We herein propose a model of institutional AI program—hospital-embedded pipelines with auditable provenance, preregistered evaluations, task-grounded tests of causal alignment and invariance, publication of failures, and local-plus-external validation—to preserve the stepwise logic of science while accepting practical opacity. Finally, the detection of failures of AI systems is not only an ethical duty but also a way to improve the systems’ performance and be considered for negotiations between AI developers and medical centers. The described canonical sequence of the scientific method remains the core scaffold of biomedical investigation. Its historical lineage is well established: Galileo Galilei integrated measurement with controlled experiment and mathematical analysis [1]; Francis Bacon formalized inductive empiricism [2]; René Descartes emphasized methodological doubt and structured reasoning [3]; Isaac Newton operationalized a hypothetic–deductive program with testable predictions grounded in mathematical laws [4]. In the nineteenth and twentieth centuries, medical science assimilated statistical design and causal inference through Pierre-Charles A. Louis’s “numerical method” [5], Ronald A. Fisher’s experimental design [6], Austin Bradford Hill’s randomized trials and causality criteria [7], and Archibald L. Cochrane’s insistence on effectiveness and efficiency in health services research [8]. Karl R. Popper’s falsificationism reframed scientific progress as the survival of hypotheses under attempted refutation [9]. The theory-leadenness of observation—as emphasized by Norwood R. Hanson [10] and later by Thomas S. Kuhn [11]—shows that data are interpreted through conceptual lenses. This insight does not entail epistemic relativism, but it does demand explicit models and reproducible tests that can be scrutinized and challenged across competing paradigms.

In AI-supported medicine, each stage has a concrete analog. “Observation” maps to data characterization (case-mix, acquisition devices, labeling protocols); the “question” to a precise construct specification (target, time horizon, population); the “hypothesis” to explicit claims about information pathways and mechanisms; “experiment(s)” to preregistered development and validation protocols; “analysis” to discrimination, calibration, clinical-subgroup performance, distribution-shift robustness, and faithfulness of explanations; “conclusions” to a risk–benefit judgment tied to a stated intended use; and “communication” to complete, reproducible artifacts (code, weights, dataset documentation) and reporting that enables critical appraisal.

Emerging domain-specific guidance now codifies these correspondences: TRIPOD-AI/TRIPOD + AI for prediction models [12], PROBAST-AI for risk of bias [13], SPIRIT-AI and CONSORT-AI for trials of AI interventions [14], and imaging-specific frameworks for medical-AI reporting [15]. Collectively, these frameworks favor prospective protocols, prespecified outcomes, and comprehensive error reporting over post hoc narratives.

Two risks deserve explicit acknowledgment. First, performance-first pipelines (e.g., end-to-end deep learning and, increasingly, foundation and large language models) can compress or skip intermediate scientific steps: a high metric is produced, and only later is an explanatory story supplied [16]. Second, explanation tools themselves require validation: saliency maps and related methods may fail basic sanity checks or be insensitive to underlying models, risking a false sense of mechanistic understanding [17–19]. Reporting standards therefore recommend evaluating “explanation fidelity” alongside predictive metrics, and medical-AI reviews now highlight dataset shift, subgroup harms, and the gap between retrospective performance and prospective clinical impact as recurring failure modes [20].

In high-stakes care, accuracy detached from mechanism and context is insufficient for inference or safe use—computers, as the remark often attributed to Pablo Picasso puts it, “are useless; they can only give you answers” [21, 22]. However, medicine requires justified questions and tested answers from which new answers come up.

“Explainable” AI is often presented as a remedy [23]. Yet current practice exposes what we define as an epistemic gap: the structural disconnect between a model’s true decision basis and the human-concept explanations that clinicians are offered. Here, three cases are proposed.

First, the decision basis may be non-human yet causally valid—for example, higher-order parenchymal texture aggregates in mammography that plausibly correlate with stromal biology without a canonical clinical label [24, 25]. Second, it may be non-human and spurious—scanner logos, view position, site-specific artifacts, or demographic proxies [26]. Third, it may be human-readable but only post hoc: coherent narratives that lack fidelity to the model’s internal reasoning. Because prevailing attribution and saliency methods can be insensitive to model or data randomization and fragile to minor perturbations, they often cannot discriminate among these cases [23, 27–29]. Consequently, face-valid heatmaps or token rationales do not, by themselves, restore the scientific steps that the black-box optimization compresses.

Clinical evidence underscores the stakes. Chest x-ray classifiers have shown apparently strong internal performance while failing to generalize across hospitals because they exploit site-specific confounders rather than pathophysiology [30]. In mammography, the same vulnerability exists: models can latch onto acquisition-related signals, vendor or site signatures, or population imbalances instead of lesion biology, yielding brittle performance across devices and screening programs [28, 31]. More broadly, shortcut learning explains why correlations that maximize training metrics can collapse under distribution shift [32]. Large language models add distinct hazards: fluent but unsupported statements and the propagation of fabricated premises [33, 34]. Although retrieval-augmented generation and conservative refusal behaviors can mitigate these risks, they do not establish mechanistic or evidential adequacy [35, 36].

Radiomics offers a parallel lesson. The field has rightly emphasized feature reproducibility as a prerequisite for clinical modeling, yet recent analyses argue that an exclusive focus on feature reproducibility can obscure informative interactions and underestimate the predictive value of features with context-dependent stability [37, 38].

In this unsettled landscape, the key deployment requirement is not universal interpretability but testability of the model’s decision basis. What matters is whether its output can be shown to rest on meaningful and reliable grounds. One dimension is causal alignment: predictions should arise from features plausibly related to disease biology or clinically relevant intermediates. This can be proved by experiments that remove or perturb candidate features, by using negative controls that should carry no information about the target, or by mediation analyses when valid surrogates exist. The second dimension is invariance: performance, and where relevant, the associated explanations, should remain stable when the data context changes, for instance, across scanners, sites, acquisition protocols, or demographic subgroups. Marked variation under such shifts could be a signal that the model is relying on shortcuts rather than clinically valid cues [27, 30, 32]. Together, these tests help determine whether non-human decision criteria reflect genuine signals or spurious artifacts, without requiring complete transparency into the model’s internal workings.

Hospital-embedded local development offers a pragmatic advantage: training and validation on the population in which the system will be used. Local data reflect the same scanners, protocols, reporting styles, and care pathways; ecological validity improves, and calibration error typically narrows. However, local does not mean unbiased: in-house pipelines can propagate site-specific measurement error and labeling conventions, underrepresent rare subgroups, and reduce portability across a regional network [32, 39]. In language tasks, institutional corpora and retrieval can align outputs with house style while risking echo-chamber effects when local norms diverge from guidelines. Local development is best treated as a bias-reshaping step that must be paired with environment-stratified external validation to preserve equity and transportability [40]. Table 1 summarizes the main benefits, risks, and potential mitigations of local development.

**Table 1:** Local training/validation on the deployment population: benefits, risks, and mitigations

Dimension	Benefit	Risk if used alone	Mitigation
Domain mismatch and calibration	Alignment with scanners, protocols, language; improved calibration	Overfitting to site idiosyncrasies	External, environment-stratified validation; periodic recalibration
Bias and equity	Reflects local case-mix and workflows	Entrances measurement/label bias; misses rare subgroups	Subgroup reporting; targeted augmentation; negative-control features
Transportability	Optimizes for local practice	Reduced portability across sites and patient mobility	Validate on external cohorts; document intended-use boundaries
Explainable AI and human factors	Explanations tuned to local readers/tasks	Echo-chamber narratives; illusion of understanding	Fidelity/stability tests; task-specific endpoints
Governance	Clear ownership of data and updates	Insular changes without scrutiny	Preregistered change control; public failure logs; TRIPOD and CONSORT-AI/SPIRIT-AI

We therefore are in favor of defining specific institutional AI policies and governance approaches that can accept practical opacity but preserve the core of the scientific method. The program begins with auditable provenance—immutable versioning of data, labels, code, and weights—so that each update is treated as a protocol amendment. Before deployment or update, institutions preregister acceptance tests that jointly cover discrimination (with uncertainty estimates), calibration on the local case-mix and on external cohorts, invariance across environments and acquisition settings, and, where explanations are used for user-facing tasks such as lesion localization, quantitative fidelity and stability of those explanations [17, 41]. After deployment, drift surveillance compares incoming data and performance to preregistered baselines; failures trigger prespecified rollback or recalibration steps. Critically, local training/validation for ecological validity is paired with external, environment-stratified audits to surface shortcuts and subgroup harms. Communication is treated as a scientific step rather than an afterthought: ablations, degraded external performance, and hallucination incidents are converted into citable institutional reports aligned with established reporting standards [12, 14, 33, 42, 43].

Transparency can be valuable, but for complex systems, testability is the stronger guarantee. The aim is not to force all models into human-readable terms; it is to determine empirically whether their decision bases are causally aligned with medicine and robust to clinically plausible shifts. Local development improves fit to practice; external audits preserve generalization and equity [39, 44, 45]. Together, they narrow the epistemic gap and keep medical AI within the contours of the scientific method: explicit hypotheses, disciplined experiments, robust analyses, bounded conclusions, and full communication—including failures that could be informative as successes.

As happens for invariance demonstration, post-market surveillance (i.e., real-world evidence) has a paramount role, as highlighted by regulations in the EU, USA, and China [46]. The detection of failures of already officially approved AI systems is, first of all, an ethical duty but also a way to improve the system’s performance. From this viewpoint, these cases should be considered for contractual negotiations between AI developers and local medical centers. A famous sentence attributed to Thomas Edison says: “Each time you fail, you have eliminated another wrong option” [47].

**Fig. 1:**
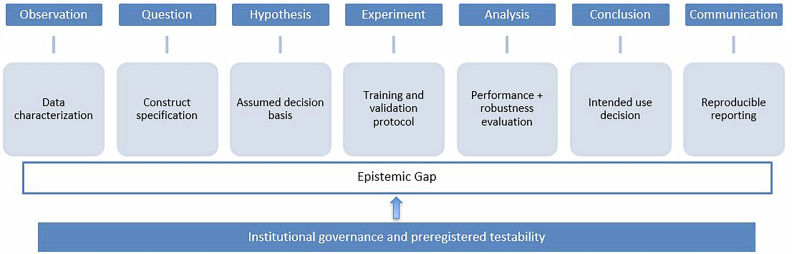
Schematic correspondence between the stages of the scientific method and the steps of medical-AI model development. The central inferential steps—hypothesis, experimental testing, and analysis—are where performance-driven pipelines may compress or bypass the justification of decision mechanisms, resulting in an epistemic gap. Institutional governance, including provenance control, preregistered acceptance tests, environment-stratified external validation, and drift surveillance, is required to re-establish testability and ensure that model outputs rest on clinically meaningful decision bases

A comprehensive graphical step-by-step scheme of the epistemic gap of AI when applied to medical imaging is offered in Fig. 1.

To summarize, we highlighted how the applications of AI in medicine—specifically in radiology—challenge the historical sequential logic of the modern scientific method. We should keep AI (and radiomics as well)—with all their peculiarities and epoch-transforming innovations—in the framework of what we know as “science”. We should fill the epistemic gap avoiding an easy approach to “explainability”—the false sense of mechanistic understanding—clearly differentiating between retrospective performance and prospective clinical impact. We should remember face-valid heatmaps or token rationales do not, by themselves, explain a model’s performance, and be ready to accept that non-human decision criteria may reflect genuine signals.

We are in favor of institutional AI policies and governance approaches that can accept practical opacity but preserve the core of the scientific method. As mentioned above, surveillance will play a pivotal role, according to the old empirical rule that “The proof of the pudding is in the eating” [48].
